# REVISE Virtual Reality Intervention to Prevent Sexual Harassment in Heterosexual Couples: Protocol for a Randomized Controlled Trial

**DOI:** 10.2196/91993

**Published:** 2026-05-14

**Authors:** Alejandro Saavedra-Roa, Pablo Vallejo-Medina, Sofia Seinfeld, Pierre Bourdin-Kreitz, Alejandro Guillén-Riquelme, Ferran Marsa-Sambola, Joan C Medina, Claudia Pineda-Marin, Clara Paz, Ariadna Angulo-Brunet, Adrian Montesano

**Affiliations:** 1 School of Psychology and Educational Sciences Universitat Oberta de Catalunya Barcelona, Catalonia Spain; 2 XR-Lab, Interdisciplinary Research and Innovation Hub Department of Computer Science, Multimedia and Telecommunication Universitat Oberta de Catalunya Barcelona, Catalonia Spain; 3 Faculty of Health Sciences Valencian International University Valencia, Valencia Spain; 4 Well-Being, Health, and Society Research Group School of Psychology and Education Universidad de Las Américas Quito, Pichincha Ecuador; 5 Measuring and Improving Student Success Futures of Education in the Digital Age Research Centre (UOC-FuturEd) Universitat Oberta de Catalunya Barcelona, Catalonia Spain

**Keywords:** sexual harassment, violence against women, virtual reality, immersive virtual reality, primary prevention, empathy

## Abstract

**Background:**

A series of studies suggests that immersive virtual reality (IVR) can enhance perspective-taking and behavioral change by simulating real-world scenarios experienced from different points of view. IVR has emerged as a promising tool for the prevention of gender-based violence, including sexual harassment. However, evidence on the effectiveness of first-person embodiment–based interventions in fostering empathy and modifying gender-related attitudes remains limited.

**Objective:**

This study aims to evaluate the efficacy of REVISE (Realidad Virtual Inmersiva y Sexualidad), an IVR-based intervention designed to influence beliefs related to sexual aggression and related psychosocial mechanisms in heterosexual men through partner identity exchange.

**Methods:**

Ninety heterosexual couples will be randomly assigned to one of three experimental conditions: (1) IVR with partner identity exchange in a sexual harassment scenario, (2) IVR with partner identity exchange in a neutral scenario, or (3) third-person observation of a sexual harassment scenario. The intervention consists of a single IVR session, with assessments conducted at baseline, postintervention, and at 3-month follow-up. Data will include self-report questionnaires, physiological measures (eye tracking and pupillometry), and semistructured qualitative interviews. The primary outcome is the change in beliefs related to sexual aggression. Secondary outcomes include empathy, gender role attitudes, relationship functioning, and physiological responses.

**Results:**

At the time of manuscript submission, recruitment had not yet begun. The study is expected to generate quantitative and qualitative data on changes in beliefs related to sexual aggression, empathy, gender-related attitudes, and physiological responses across experimental conditions.

**Conclusions:**

The REVISE trial will contribute to the evidence base on IVR interventions for the prevention of gender-based violence. By integrating embodiment, behavioral simulation, and multimodal assessment, this study may inform the development of future technology-based interventions aimed at promoting gender-equitable and prosocial behaviors.

**Trial Registration:**

ClinicalTrials.gov NCT06839937; https://clinicaltrials.gov/study/NCT06839937

**International Registered Report Identifier (IRRID):**

PRR1-10.2196/91993

## Introduction

Violence against women (VAW) is defined as any behavior that results in or is likely to result in physical, sexual, or psychological harm to a woman [1,2]. It is estimated that approximately 1 in 3 (30%) women worldwide have experienced some form of physical or sexual violence by an intimate partner—or even by a stranger—at some point in their lifetime [2]. Some of the short- or long-term consequences for survivors include posttraumatic stress, depression, anxiety, interpersonal difficulties, suicide, gynecological problems, induced abortions, unintended pregnancies, pain-related syndromes (eg, back, pelvic, or abdominal), gastrointestinal disorders, reduced mobility, increased substance abuse, smoking, and risky sexual behaviors that may facilitate sexually transmitted infections [1-5]. Additionally, VAW has broader social consequences, such as unemployment, poverty, homelessness, stigmatization, physical aggression, and femicide [1,5,6]. Some behaviors perpetrated by aggressors include humiliation, isolation, threats, and actions that restrict a woman’s autonomy [5], which often intensify over time.

Gender-based inequities have long facilitated gaps between traditional gender roles (ie, masculinity and femininity), historically privileging masculinity and, consequently, those to whom this gender has traditionally been assigned—men [7]. This disparity can lead to a lack of awareness or even a denial of inequitable gender gaps. A recent study conducted in Spain showed that men tend to minimize the impact of gender-based inequalities [8]. In fact, 3 out of 10 men believe that a partner can limit freedom and normalize jealousy as a sign of love. In addition, a considerable proportion of men (18.1%) validate controlling behaviors, such as checking their intimate partner’s cell phone [8]. Another study conducted in Mexico found that 44% of men did not perceive gender inequities [9]. These findings align with those reported by Rodríguez et al [8], where only 50.4% recognized VAW as a serious social problem in 2021. These data underscore the persistence of the sexist and traditional perspectives that shape men’s interaction with women and reinforce the rigid roles that contribute to VAW.

These numbers highlight the urgent need to intervene in traditional male perspectives to address VAW. Although some male-focused education programs designed to prevent gender-based violence focused on males have led to a decrease in physical and sexual violence, attitudes related to gender roles (eg, protection, partner control, and male provider) have proven to be particularly resistant to change [10]. This challenge is further highlighted in Ruane-McAteer et al [11,12] review, which focused on gender-transformative approaches, as it highlights the lack of high-quality interventions and the existence of inconclusive findings in relation to the efficacy of this type of intervention. Such evidence reinforces the need for robust experimental designs supported by qualitative evaluations, moving beyond psychoeducational strategies that focus solely on knowledge acquisition or attitude change [13,14]. Instead, interventions must effectively target behavioral change, ensuring that such changes can be rigorously assessed.

Virtual reality (VR) presents a promising tool for interventions targeting psychological mechanisms associated with VAW, as it facilitates ecologically valid experimental settings that allow individuals to experience realistic situations without actual exposure (ie, overcoming ethical barriers) [15,16]. Moreover, VR is recognized as a tool that can foster attitudinal, cognitive, and behavioral changes [17-19]. This methodology has been used previously and has shown effects on emotion recognition, a process potentially underlying aggressive behavior [20]. VR has also been used to replicate Milgram’s obedience experiments [21], analyze bystander response to violence [22], study intimate partner violence [23-25], and assess sexual behaviors in pedophiles [26].

Although several studies have shown the benefits of VR across different perspectives, few have explored its potential for harassment prevention through perspective-taking experiences. Of the earlier studies, one focused on increasing the realism of role-playing exercises designed to teach women how to respond effectively to harassment [27], while another aimed to develop skills for responding to sexual harassment in a simulated job interview [28]. Other studies have focused on assessing whether exposure to violence in VR has a greater impact than traditional media, but their results have been inconclusive, potentially due to limitations in evaluation methods and design [29]. However, some research suggests that VR is more effective than other approaches (eg, narrated experiences) in enhancing empathy and perspective-taking among men [30]. These findings highlight the VR’s potential as a transformative therapeutic tool for VAW prevention.

Current research has focused on whether VR exposure itself fosters changes in prosocial behaviors or whether the key factor is perceiving simulated scenarios from a first-person perspective (ie, immersive virtual reality [IVR]). Some studies have shown that virtually embodying the role of a person experiencing sexual harassment can positively influence the reduction of aggressive behaviors [30]. Therefore, the results seem to indicate that IVR could actively engage men in preventing aggressive behaviors by providing them with firsthand experiences of harassment [31], making it a valuable tool for fostering empathy and perspective-taking as a mechanism for behavioral change.

The present project examines the effectiveness of an IVR-based intervention, REVISE (Realidad Virtual Inmersiva y Sexualidad), designed to influence psychological mechanisms associated with sexual harassment and VAW. Specifically, the intervention seeks to reduce beliefs that legitimize or minimize sexual aggression while promoting empathy and perspective-taking through immersive partner identity exchange scenarios. In this intervention, male participants embody the virtual perspective of their partner in simulated harassment situations, allowing them to experience the interaction from the survivor’s viewpoint. This intervention will be designed primarily because of the critical need for a tool that

1. Incorporates a dyadic perspective: The presence of both partners in the intervention is expected to amplify the emotional impact and enhance perspective-taking due to their pre-existing emotional bond. Moreover, this is the first study to use partner embodiment rather than an anonymous avatar.
2. Target intimate partner violence: Since VAW often occurs within romantic relationships, involving both partners provides a direct and contextually relevant approach to addressing intimate partner violence.
3. Aligns with gender synchronization principles: International recommendations suggest the inclusion of men in VAW prevention.
4. Is grounded in psychological theory: REVISE includes a module that seeks to model positive partner-guided relational behaviors. This component allows behavioral changes to be fostered through a virtual experience.

## Methods

### Objectives

The primary objective of this randomized controlled trial is to evaluate the efficacy of the REVISE intervention in reducing beliefs that legitimize or minimize sexual aggression (Acceptance of Modern Myths About Sexual Aggression [AMMSA] scale) among heterosexual men at postintervention and at 3-month follow-up.

The secondary objectives are

1. To evaluate the effects of the REVISE intervention on empathy, gender role attitudes, relationship functioning, and communication patterns.
2. To assess changes in physiological responses (eye tracking and pupillometry) during the IVR experience across experimental conditions.
3. To explore participants’ subjective experiences and perceived changes following the intervention through qualitative interviews, and to integrate these findings with quantitative outcomes.

It is hypothesized that participants exposed to identity-swapping with sexual harassment scenarios will show greater reductions in beliefs legitimizing sexual aggression compared to the control conditions.

### Study Setting

This study will be conducted at the Interdisciplinary Research and Innovation Hub of the Universitat Oberta de Catalunya. This hub is 2700 square meters and is equipped with research and support staff, as well as 8 laboratories. The XR-Lab is one of the laboratories that provides the infrastructure needed to carry out the intervention sessions. It includes a monitoring system that allows the evaluation of 3 people simultaneously. Participants will be evaluated within this hub.

### Sample

Ninety heterosexual couples will participate in the study. All persons must be between the ages of 18 and 39 years, be in a relationship for at least 6 months, and give explicit consent to participate in the research, for which they must be able to read and write in Spanish. Individuals who have any physical condition that may hinder the use of the equipment (ie, epilepsy); who are currently experiencing severe psychological distress related to past harassment experiences, high levels of relationship distress, or problematic substance abuse; or who have significant mental health difficulties requiring intensive treatment will not be able to participate in the study. Participation will be encouraged through open calls on social media, where interested individuals will fill out an online form to determine their eligibility based on the inclusion and exclusion criteria. Those who meet the criteria will be invited to participate in the study. Couples who decide to participate will receive an economic compensation of €50 (US $54) at the end of the trial. No specific concomitant interventions are prohibited during participation, although participants are advised to avoid other IVR exposures during the trial period to prevent cross-contamination of experience.

**Figure 1:**
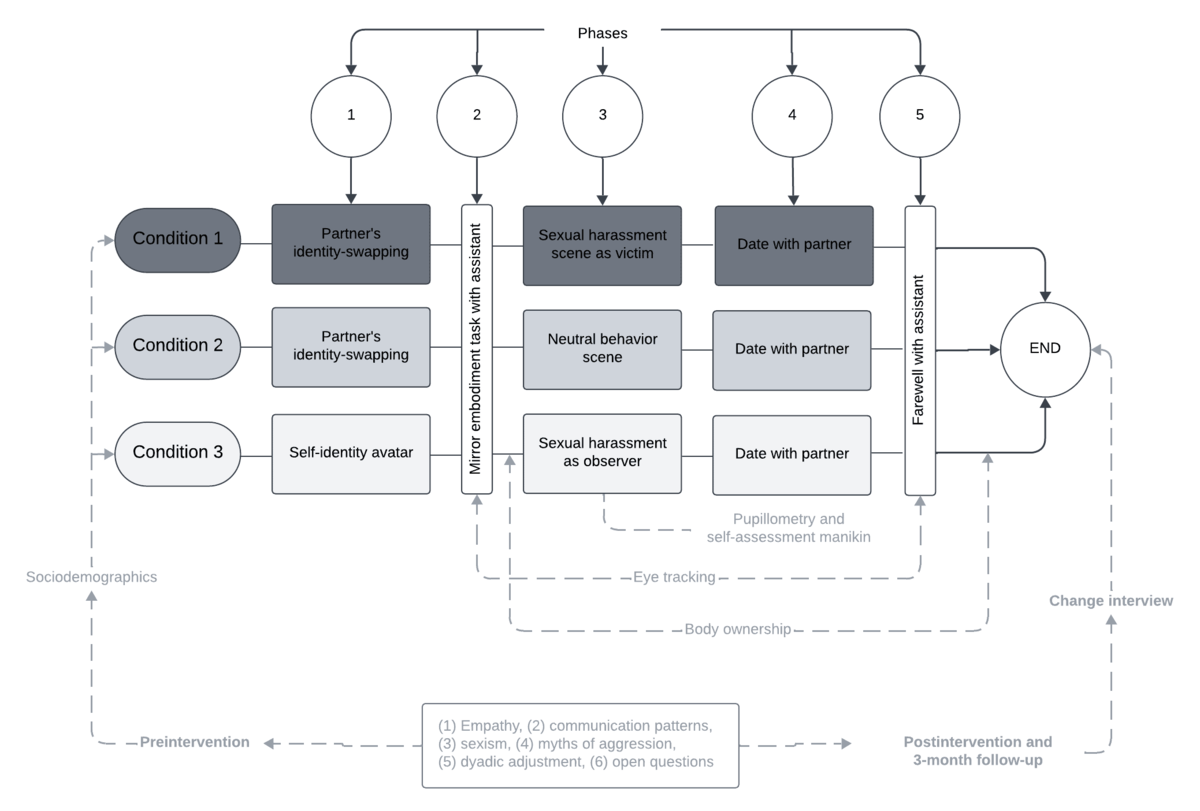
Visual outline of the experimental design. In phase 2 of the virtual reality experience, participants engage in the embodiment task.

This study is designed as a randomized, parallel-group, superiority trial with 3 arms and a 1:1:1 allocation ratio. An a priori power analysis was conducted to determine the necessary sample size to detect a meaningful effect in the primary outcome measure. Considering the study design, an estimated effect size of *d*=0.4, statistical power of 0.9, and α level of .05, the required sample size was calculated to be 84 heterosexual couples. To account for potential attrition (estimated at 7%), a total of 90 couples will be recruited. While the a priori power calculation was based on a standard framework, the final sample (N=180 individuals; 90 units of randomization and 30 couples per experimental condition) was increased to provide additional robustness for the planned longitudinal and dyadic analyses. The sample size estimation was based on a conservative effect size derived from prior studies. Although the calculation does not explicitly model the multilevel structure of the data (ie, repeated measures nested within individuals and couples), the use of mixed-effects models in the analysis phase is expected to appropriately account for this dependency structure. The allocation sequence will be computer-generated using random block sizes of 3 or 6 by a research assistant not involved in recruitment, assessment, or intervention delivery. Allocation will be concealed until participant enrollment and completion of baseline assessments (see Figure 1).

Additionally, a separate pilot study will be conducted with 10 couples, allowing for the evaluation of the performance of the application prior to full-scale implementation.

Participant recruitment will occur between May 2026 and May 2027.

### Patient and Public Involvement

Female survivors of sexual harassment and professionals working with male offenders will be involved in the cocreation of the VR scenarios used in the REVISE intervention. Their contributions will ensure that the immersive scenes are emotionally realistic, ethically appropriate, and contextually relevant for the target population. Their involvement will help refine the language, content, and narrative flow of the scenes.

### Primary Outcome

The AMMSA scale [32] will be used to assess changes in beliefs about sexual aggression (justification or minimization, subtle beliefs). This study will use a Spanish-adapted and validated version [33]. The scale consists of 30 items rated on a 7-point Likert scale ranging from 1 (“totally disagree”) to 7 (“totally agree”). Higher scores indicate greater acceptance of modern myths about sexual aggression. In a Spanish sample, the scale demonstrated high internal consistency (Cronbach α=0.91). An example item is “Alcohol is often the culprit when a man rapes a woman.”

### Secondary Outcomes

#### Changes in Empathy

We will use the Spanish-validated version of the Interpersonal Reactivity Index [34,35]. This scale evaluates the cognitive and emotional reactions of an individual to the observed experiences of another person, that is, empathy. It consists of 28 items answered on a 5-point Likert scale. We will use the Spanish-validated version [35]. The Interpersonal Reactivity Index assesses 4 different dimensions related to empathy, namely perspective taking, fantasy, empathic concern, and personal distress. Cronbach α of this index ranged from 0.68 to 0.80. An example item is “I would describe myself as a fairly soft-hearted person.”

#### Changes in Gender Role Attitudes

We will use the brief version of the Ambivalent Sexism Inventory [36], which is composed of 12 items, scaled with a 6-point Likert scale (0=strongly disagree to 5=strongly agree). It assesses sexist attitudes. We will use the Spanish brief validated scale [37], 6 items from the Hostile Sexism subscale and 6 items from the Benevolent Sexism subscale. Higher scores indicate a greater presence of sexism against women. Cronbach alphas of the scale were 0.75 and 0.81, respectively [37]. One item as an example is “Men are more willing to put themselves in danger to protect others.”

#### Changes in Couple Communication Patterns

We will use the Communication Patterns Questionnaire (CPQ) [38]. The Spanish version of the CPQ [39] will be used in this study. It is a self-report that measures the communication patterns in close relationships. It assesses 3 dimensions of communication: mutual constructive communication, communication avoidance or demand/withdrawal, and aggressive communication. The questionnaire is answered with a scale of possible use of the strategy, with 9 response options, from “very unlikely” to “very likely,” and in its current form, it is composed of 24 items. One example is “Both my partner and I blame, accuse, and criticize one another.” The Cronbach α in Spain ranged from 0.62 to 0.84.

#### Changes in Relationship Satisfaction

We will use the brief Spanish version of the Dyadic Adjustment Scale [40,41], which consists of 13 items rated on a Likert scale with variable response formats. The scale measures couple adjustment by grouping items under 3 second-order factors and a first-order structure. Scores ranged from 0 to 63, with 44 as the cutoff point to distinguish between high and low couple adjustment. Higher scores indicate better couple adjustment. For example, one item asks, “How often have you thought about divorce or separation?” with response options ranging from “never” (0) to “all the time” (5). Another item, “Do you and your partner calmly discuss any topic?” is rated from “never” (0) to “very often” (5). The scale has demonstrated good internal consistency, with a Cronbach α of 0.83.

#### Changes in Couple Flourishing

We will use the Couple Flourishing Measure [42], the Spanish version of Saavedra-Roa et al [43,44] will be used. It is a unidimensional instrument consisting of 16 items designed to be completed individually, which assesses couple flourishing using a 7-point visual analogue Likert scale. The scale ranges from 1=distressed relationship, 4=satisfied relationship, and 7=flourishing relationship. An example of an item is “Quality time spent alone with my partner is...,” where 1=monotonous (distressed), 4=pleasant (satisfied), and 7=memorable (flourishing). Scores range from 16 to 112, with higher scores indicating greater couple flourishing. The instrument demonstrated high internal consistency reliability (α=.94).

#### Change in Body Ownership Illusion

To assess body ownership and agency from a first-person perspective, a validated 4-item scale will be administered verbally at two time points during the IVR experience (see Figure 1 and Table 1):

1. Mirror embodiment phase: The assistant will ask participants to look at a virtual mirror and rate, on a scale from 0 to 10, the extent to which they felt that the virtual body reflected in the mirror was their own. Participants will then look down and respond to the same question regarding their virtual bodies.
2. Farewell phase: At the end of the IVR session, participants will answer two additional questions: “Did you feel that you owned a different body during the experiment?” and “Did you feel fear of aggression at any point in the experiment?” on the same scale (0-10).

**Table 1:** Trial schedule.

Time point	Preintervention	Allocation	Postallocation		
		0	Intervention day	1 week after intervention^a^	3 months follow-up^b^
Enrollment	✓				
Eligibility screen	✓				
Informed consent	✓				
Allocation randomization		✓			
Baseline			✓		
Embodyment task			✓		
IVR^c^ exposure condition 1			✓		
IVR exposure condition 2			✓		
IVR exposure condition 3			✓		
Self-report assessment^d^			✓	✓	✓
Physiological measures (eye-tracking and pupillometry)			✓		
Change interview			✓	✓	✓

^a^The 1-week postintervention evaluation examines short-term retention of intervention effects.

^b^The 3-month follow-up was chosen to assess long-term changes.

^c^IVR: immersive virtual reality.

^d^Self-report assessments include Interpersonal Reactivity Index (IRI), Ambivalent Sexism Inventory (ASI), Communication Patterns Questionnaire (CPQ), Acceptance of Modern Myths About Sexual Aggression (AMMSA) Scale, Dyadic Adjustment Scale (DAS), Couple Flourishing Measure (CFM), Self-Assessment Manikin (SAM), and body ownership.

#### Changes in Affective Responses

Self-Assessment Manikin (SAM) will be used [45]. This is a pictorial scale where a manikin is shown to indicate the affective reaction of a person to a given stimulus. The SAM is answered on a 9-point visual analogue scale. Valence (SAM-V), also known as pleasure, arousal (SAM-A), and dominance (SAM-D), will be used for this experiment.

#### Eye Tracking

Eye tracking is an experimental method for recording eye motion and gaze location over time and across different conditions. This is a common method of observing the allocation of visual attention [46]. In this project, it will be used to measure attention to erotic stimuli. According to sexual objectification theory, this is related to sexual aggression [47,48]. VR goggles compatible with an eye-tracking system will be used. Two different parameters will be collected: (1) number of fixations, that is, number of available fixations on the specified area of interest, which is the VR model neckline, and (2) dwell time or complete fixation time, that is, the sum of the fixation duration at the specified area (neckline) in milliseconds. In this study, the exact duration used to define a single fixation will be 100 milliseconds.

#### Pupillometry

It is the measurement of minute fluctuations in pupil diameter in response to a stimulus. Pupil dilation is thought to index autonomic activity; thus, negative stimuli trigger larger pupil dilation [49,50]. Pupillometry has recently been used to effectively evaluate the emotional effect of a nonconsented sex film clip visualization (woman as recipient) in men [50]. We will compute the pupil diameter ratio. Pupil data will be sampled at least 60 Hz, with a visual angle accuracy of 0.5. Pupil amplitude artifacts (<1 mm or >9 mm) as well as drifts and blinks will be coded as missing values [51]. As suggested, pupil data will be smoothed using a 10 Hz low-pass filter as suggested by Carvalho et al [52].

#### Change Interview

This semistructured script interview was created to assess the changes produced throughout the therapy, the useful aspects, and the adverse effects of interventions from the client’s perspective [53]. Clients are asked about the changes they have experienced with the intervention, as well as the negative changes, if any. Questions are also asked regarding the attribution of such changes and their probability of occurrence without intervention. Its functions in this study are to identify both changes that clients are aware of (in their own words), which might go unnoticed with standardized questionnaires, and adverse effects (too often neglected in research).

### Experimental Design

#### Overview

This study will seek to evaluate the efficacy of REVISE for reducing beliefs related to sexual aggression and influencing related psychological mechanisms, such as empathy and perspective-taking, among heterosexual men. The study includes 3 IVR intervention scenarios (see Figure 1). All conditions will follow the same general format: a 1-hour main session, where participants will be exposed to an IVR setting, followed by a postintervention interview to assess the impact of the experience and follow-up (for details, see Figure 1 and Table 1).

Self-reported questionnaires assessing empathy, gender attitudes, attitudes about sexual aggression, and relationship dynamics will be administered at 3 time points: baseline (preintervention), immediately postintervention, and at a 3-month follow-up. Physiological measures, including eye tracking and pupillometry, will be recorded in real time during IVR exposure to capture participants’ attention and emotional responses. Additionally, questions assessing embodiment will be administered at two key moments during the IVR session: (1) after the mirror embodiment phase and (2) at the end of the experience (farewell phase). Finally, a semistructured interview will be conducted immediately postintervention to explore participants’ subjective experiences, perceptions of the IVR scenario, and reflections on the impact of the intervention on their attitudes and beliefs related to sexual harassment and gender-based violence. The 3 experimental conditions are described in the following subsections.

#### Condition 1: Identity Exchange With Exposure to Sexual Harassment

Couples in this experimental group will experience an identity exchange with their partner in a VR environment. In this case, male participants will be exposed to an immersive scene of sexual harassment while they virtually embody the avatars of their female partners.

#### Condition 2: Identity Exchange Without Exposure to Harassment

In this condition, male participants will also virtually embody the avatar of their female partners; however, in this condition, they will go through a neutral scene, not including sexual harassment. It is intended that participants experience the perspective of their partners in a scenario that does not involve gender-based violence.

#### Condition 3: Third-Person Observer

Participants will observe a scene of sexual harassment in a virtual environment, but this time from a third-person perspective since they will embody their own avatar. The purpose will be to assess how observation of harassment affects participants’ attitudes toward sexual harassment and their empathetic responses to the survivor.

### Intervention Description

The REVISE intervention will be delivered using a head-mounted display with integrated eye-tracking and pupillometry capabilities (ie, Tobii VR headset). The virtual environments will be developed using the Unity game engine and implemented through the Tobii SDK for real-time eye-tracking and data acquisition. Avatars will be created through manual customization and synchronized with participants’ real-time body movements to induce a body ownership illusion. This embodied perspective-taking mechanism is expected to influence participants’ interpretations of sexual harassment scenarios and beliefs related to sexual aggression. During the intervention, both partners will wear head-mounted displays and interact in real time from separate physical spaces. The content and narrative structure of the virtual scenes will be identical across conditions, differing only in perspective (ie, first-person vs third-person) and exposure to sexual harassment content, according to experimental allocation.

While the primary intervention targets the male partner’s perspective, female partners play a triadic role: (1) providing ecological validity to the VR interaction through their virtual representation, (2) providing “observer-report” data on relationship functioning and perceived empathy, and (3) acting as prosocial models during the VR scenarios. Specifically, in the harassment exposure condition with identity exchange, female partners are instructed to model empathetic and assertive responses to the simulated harassment. This allows the male partner (now embodied in the female avatar) to experience and internalize a constructive, prosocial reaction to aggression from a first-person perspective. These dyadic data and interactions are essential to triangulate the self-reported changes in men and to assess whether the intervention impacts the couple’s interactional climate.

### Intervention Procedure

The intervention follows 5 phases, as shown in Figure 1. Each participant will arrive at the laboratory and provide their consent to participate in the study. They will be randomly assigned; participants will be naïve to the study hypotheses. While avatar creation takes place, the couple will answer preintervention questionnaires. Phase 1 also involves the process of personification of the avatar according to the condition in which they will be assigned (see the section on the Intervention Description).

During phase 2, the participants will perform a mirror embodiment task to familiarize themselves with the virtual environment and their new virtual body. More specifically, a female virtual assistant will welcome the participants and instruct them to perform some physical body movements while observing themselves in a virtual mirror. The observation of real-time movements in the embodied avatar, which corresponds to the participants’ real body movements, will allow for greater immersion, as well as enhance body ownership and agency toward the virtual body. For assessment purposes, the female assistant will be a sexualized avatar. During this initial outreach, eye-tracking data will be collected from the participants in relation to the assistant’s cleavage, as gaze fixation on sexualized body parts has been identified as a marker of sexual objectification [47,52]. Once the training and embodiment process is completed, participants will be asked the first questions related to embodiment to check the degree of embodiment achieved.

In phase 3, men will have a 180-second adaptation period in which they will be in an empty bar, and nothing will happen, while women will be in a neutral playful setting in all 3 conditions. Condition 1 men will be involved in a situation of sexual harassment by a group of other men (eg, unwanted flirting, verbal intimidation, and sexist comments). In condition 2, they will stay the same length of time as in the other conditions, but in this case, they will not be sexually harassed. Finally, those in condition 3 will witness the same harassment as in condition 1, but from a bystander perspective, that is, the assaulted person will be a random virtual woman. While people are exposed to the scenarios, their emotional arousal will be assessed by pupillometry in the 3 experimental conditions; however, SAM will be used at the end of phase 3.

Phase 4 will be similar for the 3 conditions. In condition 1, men will have a virtual meeting with their partners (both still with the exchanged identity) outside the context in which the harassment situation occurred. The man will be asked to recount his experience related to the harassment experienced virtually to his partner, who will then be asked to respond empathetically. In condition 2, participants will also meet with their partners (maintaining the virtual identity exchange) outside the scenario in which they were and will be asked to discuss their concerns and expectations of the relationship related to gender issues. In condition 3, participants will discuss the sexual harassment they witnessed in the bar scene, while embodying their own avatar (not exchanged). This discussion will emphasize their reflections on the observed behavior and its consequences, promoting personal insights from their perspective as bystanders.

In phase 5, the sexualized female avatar will appear to perform the farewell, and the participants’ gaze will be traced. Before the end, two questions related to body belonging will be asked. Outcome assessments will be conducted immediately after the intervention (posttest) and at 3 months (follow-up).

The intervention will be discontinued for a participant if they experience severe cybersickness (eg, nausea, dizziness, and disorientation), acute psychological distress, or technical failures that compromise the integrity of the experience or data collection. Participants will be informed that they may request to stop the session at any time without providing a reason. In such cases, the session will be immediately terminated, and appropriate support will be offered if needed.

### VR Scenes Development

Although the structure of the scenes has already been designed, they will be tested and validated through focus groups with female survivors of sexual harassment and professionals working with male aggressors. Additionally, the entire tool will be piloted with the 10 couples mentioned above.

### Trial Design

This randomized superiority trial seeks to compare the efficacy of the REVISE intervention in influencing beliefs related to sexual aggression and associated psychosocial mechanisms, including empathy and perspective-taking, among heterosexual men under three conditions: (1) partner identity exchange in a virtual sexual harassment context, (2) partner identity exchange in a neutral context (ie, no sexual violence included), and (3) no identity exchange with observation of sexual violence context. REVISE involves an accessible digital modality that allows for the immersion and experience of the scenarios to which participants will be exposed. The 3-arm design was chosen to disentangle the effects of embodiment from exposure to sexual harassment content. Condition 1 combines first-person embodiment through partner identity exchange with exposure to a sexual harassment scenario. Condition 2 controls for embodiment without harassment exposure. Condition 3 controls for harassment exposure without embodiment, as participants observe the scenario from a third-person perspective. This structure allows identification of whether observed effects on beliefs and attitudes are driven primarily by embodiment, by narrative exposure, or by their combination.

### Data Analysis Plan

All statistical analyses will be performed using R (R Core Team) with relevant packages including *lme4*, *nlme*, *geepack*, and *mice* [54].

#### Quantitative Analysis

##### Primary Analysis

The primary outcome is the change in beliefs related to sexual aggression. The primary comparison of interest is the condition × time interaction—specifically, whether change from baseline to postintervention and from baseline to 3-month follow-up differs across the 3 experimental conditions (IVR-based intervention, control condition A, and control condition B). This will be estimated using a single prespecified linear mixed-effects model with fixed effects for condition (3 levels, dummy-coded), time (3 levels, with baseline as reference), and their interaction, and random intercepts for couples and for participants nested within couples. The female partner’s scores (eg, Dyadic Adjustment Scale and Couple Flourishing Measure) will be included as covariates to control for baseline relationship quality. This model structure was selected as the primary analytic approach; ANOVA-based omnibus tests and simpler regression models will not serve as primary analyses but may be reported descriptively.

##### Couple-Level Dependence

Because participants are recruited and randomized as couples, observations within a dyad are nonindependent. This clustering is addressed through the inclusion of couple-level random effects in all mixed-effects models. As a sensitivity analysis, generalized estimating equations with an exchangeable working correlation structure (*geepack*) will also be estimated to obtain population-average intervention effects.

##### Secondary Outcomes

Empathy, gender attitudes, relationship functioning, and physiological responses will each be analyzed using prespecified mixed-effects models with the same fixed-effects structure as the primary model, adapted to the distribution of each outcome (eg, binomial models for binary outcomes and Poisson or negative binomial for count outcomes). Eye-tracking and pupillometry measures collected during IVR exposure will be analyzed using linear mixed-effects models with random intercepts for participant and, where applicable, for stimulus item. Descriptive statistics and effect sizes (Cohen *d* or partial *η*²) with 95% CIs will be reported for all outcomes.

##### Missing Data

Missing outcome data will be handled using multiple imputation by chained equations (MICE, *mice* package), generating 50 imputed datasets combined using Rubin’s rules. Imputation models will include all analysis variables, auxiliary variables predictive of missingness, and couple-level identifiers to respect the clustered structure. Results from imputed analyses will be reported alongside complete-case sensitivity analyses. The assumption of “missing at random” will be evaluated through pattern analysis and tested in sensitivity analyses under a “missing not at random” scenario using pattern-mixture models. All analyses will follow an intention-to-treat principle.

#### Qualitative Analysis

Qualitative data from postintervention interviews and open-ended questions will be analyzed using reflexive thematic analysis. This approach allows identification of patterns in participants’ experiences, perceived changes, and interpretations of the intervention. The analysis will involve systematic coding of transcripts, iterative development of themes, and collaborative interpretation among members of the research team.

Exploratory computational text analysis (eg, co-occurrence networks and sentiment analysis using *pysentimiento* and *syuzhet* [55-58]) may be conducted as a supplementary descriptive tool to explore linguistic patterns within the corpus but will not replace the primary thematic analysis.

### Governance

The coordinating center for this trial is the Universitat Oberta de Catalunya, led by the corresponding author. The research team includes clinical psychologists, VR developers, and data analysts. While no independent steering committee or endpoint adjudication committee is designated due to the study’s minimal-risk and noninvasive nature, weekly team meetings will ensure trial fidelity, recruitment flow, and protocol adherence. No data monitoring committee has been established for this trial, as the intervention is noninvasive, the anticipated risks are minimal, and the study duration is short, making formal safety monitoring beyond standard institutional oversight unnecessary. No interim analyses or formal stopping guidelines are planned, given the short duration of the study and the absence of anticipated serious risks. Likewise, no independent auditing procedures are planned beyond standard institutional oversight, as the trial is conducted within an academic setting and does not involve pharmacological or invasive interventions. No specific compensation arrangements for trial-related harm are foreseen, as no physical harm is expected; however, participants will be informed of their right to withdraw at any time and will be provided with appropriate support in case of discomfort.

### Ethical Considerations

This study was conducted following the principles of the 1975 Declaration of Helsinki, as revised in 1983, by the Research Ethics Committee of the Universitat Oberta de Catalunya. Only the principal investigators and authorized members of the research team will have access to the final trial dataset. This access will be strictly regulated to ensure compliance with ethical guidelines and data protection regulations. All participants will give consent to participate voluntarily in the study, and the ethics committee has already evaluated the procedure for obtaining their consent. This study received approval from the ethics committee of the university, with minutes of approval CE23-PF02. Given the noninvasive nature of the intervention, no formal adverse event monitoring is planned. However, participants may report any discomfort or emotional distress to the research team, and referrals to psychological support will be provided if necessary. No posttrial care is planned. The intervention poses minimal risk, and no compensation mechanism for trial-related harm is required.

Participants will receive financial compensation for their participation in the study. Specifically, each couple will receive €50 (US $54) upon completion of the study procedures. This compensation is intended to acknowledge participants’ time and involvement and is not contingent on performance or study outcomes.

The results of this study will be disseminated through publication in peer-reviewed journals and presentations at academic conferences and scientific meetings. Only aggregated data will be reported to ensure participant confidentiality. The full protocol, deidentified datasets, and analysis code will be available upon reasonable request to the corresponding author after trial completion and publication.

## Results

As of January 2026, the trial has not started recruiting participants. Ethical approval has been obtained (approval code CE23-PF02), and the intervention materials and procedures are finalized for implementation. Recruitment and data collection are planned from March 2026 to March 2027, with primary endpoint assessments conducted immediately postintervention and at 3-month follow-up. The first results are expected after completion of follow-up assessments.

## Discussion

### Principal Findings

This study aims to evaluate the potential of IVR as a tool for influencing beliefs related to sexual aggression and associated psychosocial mechanisms among heterosexual men. The REVISE intervention is designed to simulate harassment scenarios from a first-person perspective through partner identity exchange, allowing participants to experience situations from the perspective of their partner. It is expected that this embodied perspective-taking experience will lead to reductions in beliefs that legitimize or minimize sexual aggression, as measured by the AMMSA scale. Additionally, changes may be observed in related psychosocial constructs such as empathy, gender role attitudes, and relationship functioning.

It is expected that the intervention will produce stronger effects in the condition involving partner identity exchange combined with exposure to a harassment scenario, as this condition maximizes embodied perspective-taking. More moderate effects may emerge in the identity exchange condition without harassment exposure, particularly for relational variables such as empathy and partner-related outcomes. In contrast, the third-person observation condition may produce more limited changes because participants do not experience the scenario through an embodied perspective.

Previous research has suggested that IVR can facilitate perspective-taking and emotional engagement by placing individuals in simulated environments that resemble real-world social situations [17-19]. Experimental studies have shown that embodying a virtual avatar from a first-person perspective can influence empathy, bias reduction, and attitudes toward others [20,30]. However, relatively few studies have explored the potential of immersive VR interventions for addressing attitudes associated with sexual harassment and gender-based violence, particularly within dyadic contexts. By incorporating partner identity exchange and relational interaction within the virtual scenario, the REVISE intervention extends prior work by exploring how relational dynamics may influence perspective-taking processes and attitude change [30,31].

### Strengths and Limitations

This study has several strengths. First, it employs a randomized controlled design with 3 experimental conditions, allowing differentiation between the effects of embodiment and exposure to harassment-related content. Second, the study integrates multiple sources of data, including self-report measures, physiological indicators, and qualitative interviews, providing a multimodal assessment of the intervention’s effects. Third, the dyadic design allows examination of relational processes that may shape responses to simulated harassment scenarios.

At the same time, several limitations should be acknowledged. The study focuses on short-term changes in beliefs and attitudes rather than direct behavioral outcomes. Although these constructs are theoretically linked to behavioral responses, the trial cannot directly demonstrate reductions in harassment behaviors in the general population. Furthermore, since the primary outcomes are based on self-reported empathy and attitudes, the results should be interpreted as changes in psychological predispositions rather than observed behavioral conduct. Additionally, the sample consists of heterosexual couples recruited from the general population, which may limit the generalizability of the findings to other populations or relationship structures. Regarding the female participants, their involvement is crucial for the ecological validity of the dyadic interaction. The study design minimizes participant burden by using noninvasive measures, and potential distress is mitigated through strict exclusion criteria for couples in high-conflict relationships and the provision of psychological debriefing if needed. Finally, the intervention involves a single VR session, and longer-term behavioral effects cannot be assumed.

### Future Directions

If the intervention demonstrates promising effects on beliefs and attitudes related to sexual aggression, future research may explore the adaptation of similar VR-based tools for broader educational and prevention programs. Further studies could also investigate the application of immersive perspective-taking interventions in different populations, including educational settings, bystander intervention programs, or rehabilitation contexts.

## Appendix

Multimedia Appendix 1 Peer-reviewer report from Proyectos de Generación de Conocimiento 2022, Ministerio de Ciencia e Innovación (Spain).

Multimedia Appendix 2 SPIRIT checklist.

## Abbreviations

AMMSA: Acceptance of Modern Myths About Sexual Aggression Scale
CPQ: Communication Patterns Questionnaire
IVR: immersive virtual reality
MICE: multiple imputation by chained equations
REVISE: Realidad Virtual Inmersiva y Sexualidad
SAM: Self-Assessment Manikin
VAW: violence against women
VR: virtual reality

## Data Availability

The datasets generated and/or analyzed during the study will be available from the corresponding author on reasonable request.

## References

[1] (2021). Violence against women prevalence estimates, 2018. World Health Organization.

[2] (2024). Violence against women. World Health Organization.

[3] Elvin-Nowak YM, Backman-Enelius MM, Jonas WC, Eriksson JA, Åhlund DS, Barimani MM (2023). Intimate partner violence and negative health consequences: a cross-sectional study among women in a regional sample in Sweden. Scand J Public Health.

[4] Forciniti A, Zavarrone E (2023). Data quality and violence against women: the causes and actors of femicide. Soc Indic Res.

[5] Tourné García M, Herrero Velázquez S, Garriga Puerto A (2024). Consecuencias para la salud de la violencia contra la mujer por la pareja [Health consequences of violence against women by the couple]. Aten Primaria.

[6] Gunarathne L, Bhowmik J, Apputhurai P, Nedeljkovic M (2023). Factors and consequences associated with intimate partner violence against women in low- and middle-income countries: a systematic review. PLoS One.

[7] Klein D (2016). Violence against women: some considerations regarding its causes and its elimination. Crime Delinq.

[8] Rodríguez E, Calderón D, Kuric S, Sanmartín A (2021). Barómetro Juventud y Género 2021: Identidades, Representaciones y Experiencias en una Realidad Social Compleja [in Spanish].

[9] Álvarez-Aguilar N, Habib-Mireles L, García-Ancira C (2022). Gender perceptions of women and men of engineering career: similarities and differences. Rev Univ Soc.

[10] Marcus R (2018). Programming to Promote Gender-Equitable Masculinities Among Adolescent Boys: Key Findings From a Rigorous Review.

[11] Ruane-McAteer E, Amin A, Hanratty J, Lynn F, Corbijn van Willenswaard K, Reid E, Khosla R, Lohan M (2019). Interventions addressing men, masculinities and gender equality in sexual and reproductive health and rights: an evidence and gap map and systematic review of reviews. BMJ Glob Health.

[12] Ruane-McAteer E, Gillespie K, Amin A, Aventin A, Robinson M, Hanratty J, Khosla R, Lohan M (2020). Gender-transformative programming with men and boys to improve sexual and reproductive health and rights: a systematic review of intervention studies. BMJ Glob Health.

[13] Bell C, Coates D (2022). The Effectiveness of Interventions for Perpetrators of Domestic and Family Violence: An Overview of Findings From Reviews.

[14] DeGue S, Valle LA, Holt MK, Massetti GM, Matjasko JL, Tharp AT (2014). A systematic review of primary prevention strategies for sexual violence perpetration. Aggress Violent Behav.

[15] Dobbin F, Kalev A (2019). The promise and peril of sexual harassment programs. Proc Natl Acad Sci USA.

[16] Madary M, Metzinger TK (2016). Recommendations for good scientific practice and the consumers of VR-Technology. Front Robot AI.

[17] Maister L, Slater M, Sanchez-Vives MV, Tsakiris M (2015). Changing bodies changes minds: owning another body affects social cognition. Trends Cogn Sci.

[18] Riva G, Baños RM, Botella C, Mantovani F, Gaggioli A (2016). Transforming experience: the potential of augmented reality and virtual reality for enhancing personal and clinical change. Front Psychiatry.

[19] Slater M, Sanchez-Vives MV (2016). Enhancing our lives with immersive virtual reality. Front Robot AI.

[20] Seinfeld S, Arroyo-Palacios J, Iruretagoyena G, Hortensius R, Zapata LE, Borland D, de Gelder B, Slater M, Sanchez-Vives MV (2018). Offenders become the victim in virtual reality: impact of changing perspective in domestic violence. Sci Rep.

[21] Slater M, Antley A, Davison A, Swapp D, Guger C, Barker C, Pistrang N, Sanchez-Vives MV (2006). A virtual reprise of the Stanley Milgram obedience experiments. PLoS One.

[22] Jouriles EN, Kleinsasser A, Rosenfield D, McDonald R (2016). Measuring bystander behavior to prevent sexual violence: moving beyond self reports. Psychol Violence.

[23] Gonzalez-Liencres C, Zapata LE, Iruretagoyena G, Seinfeld S, Perez-Mendez L, Arroyo-Palacios J, Borland D, Slater M, Sanchez-Vives MV (2020). Being the victim of intimate partner violence in virtual reality: first-versus third-person perspective. Front Psychol.

[24] Seinfeld S, Zhan M, Poyo-Solanas M, Barsuola G, Vaessen M, Slater M, Sanchez-Vives MV, de Gelder B (2021). Being the victim of virtual abuse changes default mode network responses to emotional expressions. Cortex.

[25] Trottier D, Goyette M, Benbouriche M, Renaud P, Rouleau J, Bouchard S, Rizzo S, Bouchard AS (2019). Using virtual reality with child sexual offenders: assessing deviant sexual interests. Virtual Reality for Psychological and Neurocognitive Interventions.

[26] Jouriles EN, McDonald R, Kullowatz A, Rosenfield D, Gomez GS, Cuevas A (2009). Can virtual reality increase the realism of role plays used to teach college women sexual coercion and rape-resistance skills?. Behav Ther.

[27] Sadeh-Sharvit S, Giron J, Fridman S, Hanrieder M, Goldstein S, Friedman D, Brokman S (2021). Virtual reality in sexual harassment prevention: proof-of-concept study. Proceedings of the 21st ACM International Conference on Intelligent Virtual Agents.

[28] Steinfeld N (2019). To be there when it happened: immersive journalism, empathy, and opinion on sexual harassment. Journal Pract.

[29] Ventura S, Cardenas G, Miragall M, Riva G, Baños R (2021). How does it feel to be a woman victim of sexual harassment? The effect of 360°-video-based virtual reality on empathy and related variables. Cyberpsychol Behav Soc Netw.

[30] Neyret S, Navarro X, Beacco A, Oliva R, Bourdin P, Valenzuela J, Barberia I, Slater M (2020). An embodied perspective as a victim of sexual harassment in virtual reality reduces action conformity in a later Milgram obedience scenario. Sci Rep.

[31] Sivermo F, Wallinius M, Anckarsäter H (2025). A pilot study on treatment content in virtual reality-assisted aggression therapy at a maximum-security forensic psychiatric clinic. Sci Rep.

[32] Gerger H, Kley H, Bohner G, Siebler F (2007). The acceptance of modern myths about sexual aggression scale: development and validation in German and English. Aggress Behav.

[33] Megías JL, Romero-Sánchez M, Durán M, Moya M, Bohner G (2011). Spanish validation of the Acceptance of Modern Myths about Sexual Aggression Scale (AMMSA). Span J Psychol.

[34] Davis MH (1980). A multidimensional approach to individual differences in empathy. JSAS Cat Sel Doc Psychol.

[35] Pérez-Albéniz A, De Paúl J, Etxeberría J, Montes MP, Torres E (2003). Adaptación del Interpersonal Reactivity Index (IRI) al español. Psicothema.

[36] Glick P, Fiske ST (1997). Hostile and benevolent sexism: measuring ambivalent sexist attitudes toward women. Psychol Women Q.

[37] Castro YR, Fernández ML, Fernández MVC (2009). Validación de la versión reducida de las escalas ASI y AMI en una muestra de estudiantes españoles. Psicogente.

[38] Christensen A, Sullaway M (1984). Communication patterns questionnaire. J Marriage Fam.

[39] Montes-Berges B (2009). Patrones de comunicación, diferenciación y satisfacción en la relación de pareja: validación y análisis de estas escalas en muestras españolas. Anal Psicol.

[40] Spanier GB (1976). Measuring dyadic adjustment: new scales for assessing the quality of marriage and similar dyads. J Marriage Fam.

[41] Santos-Iglesias P, Vallejo-Medina P, Sierra J (2009). Propiedades psicométricas de una versión breve de la escala de ajuste diádico en muestras españolas. Int J Clin Health Psychol.

[42] Sanri AG, Halford WK, Rogge RD, von Hippel W (2021). The couple flourishing measure. Fam Process.

[43] Saavedra-Roa A, Pineda-Marín C, Vallejo-Medina P, Montesano A (2026). Adaptation and validation of the couple flourishing measure among Spaniards. (forthcoming).

[44] Saavedra-Roa A, Rodríguez-González M, Pineda-Marín C, Vallejo-Medina P, Montesano A (2026). Clinical validity of the couple flourishing measure in a Spanish population: a comparative study with the dyadic adjustment scale. (forthcoming).

[45] Bradley MM, Lang PJ (1994). Measuring emotion: the self-assessment manikin and the semantic differential. J Behav Ther Exp Psychiatry.

[46] Borys M, Plechawska-Wójcik M (2017). Eye-tracking metrics in perception and visual attention research. Eur J Med Technol.

[47] Hollett RC, Rogers SL, Florido P, Mosdell B (2022). Body gaze as a marker of sexual objectification: a new scale for pervasive gaze and gaze provocation behaviors in heterosexual women and men. Arch Sex Behav.

[48] Bareket O, Shnabel N, Abeles D, Gervais S, Yuval-Greenberg S (2019). Evidence for an association between men’s spontaneous objectifying gazing behavior and their endorsement of objectifying attitudes toward women. Sex Roles.

[49] Laeng B, Sirois S, Gredebäck G (2012). Pupillometry: a window to the preconscious. Perspect Psychol Sci.

[50] Babiker A, Faye I, Malik A (2013). Pupillary behavior in positive and negative emotions. 2013 IEEE International Conference on Signal and Image Processing Applications.

[51] Kawai T, Hirahara M, Tomiyama Y, Atsuta D, Häkkinen J (2013). Disparity analysis of 3D movies and emotional representations. Stereoscopic Displays and Applications XXIV.

[52] Carvalho J, Rosa PJ, Pereira B (2021). Dynamic risk factors characterizing aggressive sexual initiation by female college students. J Interpers Violence.

[53] Elliott R, Slatick E, Urman M, Frommer J, Rennie DL (2001). Qualitative change process research on psychotherapy: alternative strategies. Qualitative Psychotherapy Research: Methods and Methodology.

[54] R Core Team (2024). R: A Language and Environment for Statistical Computing.

[55] Saavedra-Roa A, Pineda-Marín C, Vallejo-Medina P, Montesano A (2026). Exploring transformer-based sentiment analysis in affective and sexual relationship dynamics: pysentimiento as a tool. (forthcoming).

[56] García-Vega M, Díaz-Galiano MC, García-Cumbreras MA, Del FM, Montejo-Raéz A, Jiménez-Zafra SM (2020). Overview of TASS 2020: introducing emotion detection. https://ceur-ws.org/Vol-2664/tass_overview.pdf.

[57] Pérez JM, Furman DA, Alemany LA, Luque F (2022). RoBERTuito: a pre-trained language model for social media text in Spanish. arXiv. Preprint posted online.

[58] Pérez JM, Rajngewerc M, Giudici JC, Furman DA, Luque F, Alemany LA, Martínez MV (2024). pysentimiento: a Python toolkit for opinion mining and social NLP tasks. arXiv. Preprint posted online.

