# Supplementary material for: REVISE Virtual Reality Intervention to Prevent Sexual Harassment in Heterosexual Couples: Protocol for a Randomized Controlled Trial
Source: JMIR Res Protoc. 2026 May 14;15:e91993. doi: 10.2196/91993 (PMC13219989; doi:10.2196/91993)
Supplement: Multimedia Appendix 1 [file resprot_v15i1e91993_app1.pdf]

MINISTERIO DE CIENCIA E INNOVACIÓN

AGENCIA ESTATAL DE INVESTIGACIÓN

Proyectos de Generación de Conocimiento 2022<br>Investigación Orientada Tipo B	
**Referencia:**	PID2022-141198OB-I00
**Área:**	Psicología
**Subárea:**	Psicología
**Investigador/a principal**	MONTESANO DEL CAMPO, ADRIAN VALLEJO MEDINA / PABLO
**Título:**	INTERVENCION DE REALIDAD VIRTUAL PARA PAREJAS EN LA PREVENCION DE LA VICTIMIZACION SEXUAL: UN ENFOQUE TRANSFORMADOR DE INTERCAMBIO DE GENERO

De acuerdo con los principios de discrecionalidad técnica e independencia, este informe de valoración científico técnica se elabora teniendo en cuenta el consenso adoptado en la comisión técnica, una vez analizadas las valoraciones cualitativas de los informes de experto y las opiniones de los expertos de la comisión, y a la vista del conjunto de proyectos presentados a cada área temática. Por ello, este informe no tiene por qué asumir, en todos sus términos, las valoraciones aportadas por los expertos en sus informes, que pueden ser discrepantes y que se reconocen inevitables y legítimas siempre que no incurran en errores técnicos o fácticos graves y manifiestos.

PROYECTOS DE GENERACIÓN DE CONOCIMIENTO 2022 MODALIDAD: INVESTIGACIÓN ORIENTADA TIPO B

INFORME DE VALORACIÓN CIENTÍFICO TÉCNICA - COMISIÓN TECNICA

PARTE 1

CRITERIOS DE EVALUACIÓN

1.Calidad y viabilidad de la propuesta

UMBRAL 30

Puntuación de 0 a 40: 34.99

1.1. Calidad de la propuesta

Según la Comisión Científico Técnica, el proyecto presentado es una propuesta en el ámbito de la prevención de la victimización sexual. La propuesta presenta un alto grado de originalidad en su hipótesis, con objetivos relevantes, claros y realistas, y metodología muy novedosa y adecuada ya que utiliza realidad virtual para cambiar las actitudes que ejercen acoso sexual sobre las mujeres Además, el proyecto presentado es una propuesta con una buena contribución y adecuación al reto seleccionado de salud y bienestar.

Puntuación 0 a 25: 21.87

1.2 Viabilidad de la propuesta

En opinión de la Comisión Científico Técnica, la viabilidad de la propuesta es muy buena, lo cual viene avalado por las publicaciones previas del equipo en esta temática. Tanto las actividades propuestas como de los recursos humanos, materiales y de infraestructura con las que cuenta el equipo son adecuados para alcanzar los objetivos del proyecto. Como posibles mejoras, la CCT indica que se debería especificar mejor cómo se van a reclutar las muestras, especialmente, como se va a tener acceso al historial de acoso de los participantes.

Puntuación 0 a 15: 13.12

2. Calidad y trayectoria de los componentes del proyecto

UMBRAL 20

El equipo de investigación ha realizado buenas contribuciones científico-técnicas dentro de su área de la aplicación de la eHealth. La experiencia tanto de los IP como de los miembros del equipo es muy buena para llevar a cabo el proyecto y los resultados previos del equipo en la temática del proyecto son muy buenos. Se echa en falta una mayor internacionalización de los IPs, aunque en el equipo se han incorporado investigadores internacionales. La Comisión Científico Técnica ha priorizado el proyecto para optar a un contrato predoctoral para la formación de doctores, dada la valoración alcanzada y el plan de formación presentado.

Puntuación 0 a 30: 23.25

3. Impacto científico, económico y social esperado de los resultados

Los resultados de esta propuesta darán lugar a un avance importante en el conocimiento científico-técnico dentro del ámbito del acoso sexual que sufren las mujeres. El plan de divulgación científica de los mismos está bien elaborado y es plausible que publiquen los seis+un artículo que indican en la memoria. En cuanto al impacto social y económico de sus resultados, la propuesta tendrá un impacto social bueno ya que desarrollará una herramienta que podrá ser utilizada para la prevención del acoso sexual. Presenta un plan de difusión de resultados claro y realista que incluye la elaboración de una página web o podcast para la difusión la población general. También cuentan con el apoyo de instituciones públicas y asociaciones de Barcelona interesadas en los resultados del proyecto y la herramienta que pretenden desarrollar

UMBRAL 10

Puntuación 0 a 20: 17.5

4. Adecuación del presupuesto asociado

En relación al presupuesto, existen algunas partidas insuficientemente justificadas, como pueden ser las de personal o la de asistencia a congresos. En resumen, la Comisión Científico Técnica ha hecho una valoración positiva de este proyecto, destacando varios aspectos positivos por los que cabría de esperar que los resultados obtenidos serán valiosos y contribuirán al avance del conocimiento científico-técnico. Teniendo en cuenta todas estas consideraciones y el carácter competitivo de la convocatoria y las disponibilidades presupuestarias, se propone la financiación de este proyecto.

Puntuación 0 a 10: 7.63

Valoración Global

Puntuaciónde 0 a 100: 83.37

PARTE 2

OTROS ASPECTOS A CONSIDERAR

a) Aspectos relacionados con zonas polares o campañas oceanográficas(Cumplimentar solo en los proyectos que proceda)

na

b) Condiciones específicas para la ejecución de determinados proyectos (Cumplimentar solo en los proyectos con aspectos relacionados con las condiciones o implicaciones recogidas en el Anexo IV de la convocatoria).

na

INFORME-EXPERTO 1

PARTE 1

CRITERIOS DE EVALUACIÓN

1.Calidad y viabilidad de la propuesta

1.1 Calidad de la propuesta

El proyecto que se somete a evaluación se incluye dentro de los Proyectos de investigación orientada, tipo B, dentro del área temática principal de Psicologia y de la subárea temática de estudios feministas, de las mujeres y de género. Con el título ¿Intervención de realidad virtual para parejas en la prevención de la victimización sexual: un enfoque transformador de intercambio de género¿ se programa para un periodo de 4 años y tiene como objetivo aumentar las herramientas disponibles para la prevención de la victimización sexual mediante el desarrollo y la evaluación de la eficacia de REVISE (REalidad Virtual Immersiva y SExualidad), una aplicación de Realidad Virtual (RV) que permite a los hombres cambiar de género y experimentar el acoso sexual desde la perspectiva de una víctima. Hipótesis principal de este proyecto es probar una novedosa intervención de RV de intercambio de identidad de pareja, REVISE, que se espera que tenga un significativo impacto en el comportamiento de los hombres y en su comprensión crítica de la violencia sexual y género hacia las mujeres. Se espera que la perspectiva de experimentar a través de la RV el impacto que sufre la víctima de violencia sexual mejore la empatía de los participantes y reduzca las actitudes sexistas basadas en el género en términos de normas estereotipadas nocivas de masculinidad y comportamiento sexistas. lideran este proyecto dos IPs hombres, teniendo en cuanta que el proyecto aborda la violencia sexual ejercida contra mujeres hubiera sido mas adecuado la inclusión de una IP mujer especialista en la temática.

1.2 Viabilidad de la propuesta

Los objetivos están planteados con claridad y precisión, centrados en evaluar la eficacia de REVISE con una muestra de 90 parejas heterosexuales jóvenes, considerando que este tamaño de la muestra permite llevar a cabo los análisis planteados para cumplir los objetivos del estudio. Se llevará a cabo una estrategia de análisis de método mixto, que incluye administración de cuestionarios y medidas fisiológicas como seguimiento ocular y pupilometría durante la experiencia de RV. También se llevará a cabo un análisis longitudinal para comprender el impacto a largo plazo de la intervención, utilizando modelos de efectos mixtos y modelos lineales generalizados con R. El proyecto también incluye técnicas de minería de datos como co-redes de ocurrencia y análisis de sentimiento.

2. Calidad y trayectoria de los componentes del proyecto

Los IPs de este proyecto han desarrollado líneas de investigación diferentes y este es el primer proyecto de colaboración. El IP1 ha desarrolla una línea de investigación en realidad virtual y pertenece al grupo de intervención en psicología clínica y de la salud y promoción del bienestar (SGR2021), está interesado en eHealth especialmente en el uso de la RV para mejorar la salud y promover la calidad de vida y de las relaciones. Ha publicado 39 artículos en revistas indexadas en JCR. El IP2 ha desarrollado mayoritariamente su línea de investigación en el ámbito de la sexualidad, en menor medida sobre la violencia sexual. El equipo de investigación incorpora a 5 integrantes, dentro de los que se integra a un ingeniero informático que ha trabajado en realidad virtual y una psicóloga que también ha trabajado en RV. En el equipo de trabajo 6 incorporaciones entre las que destacan investigadores de universidades extranjeras. Los miembros del equipo no han colaborado previamente, lo que muestra que no conforman un equipo consolidado sino una sinergia de investigadores que se unen para desarrollar la propuesta que se somete a evaluación.

3. Impacto científico, económico y social esperado de los resultados

Impacto científico Derivado de los conocimientos relativos a los procesos psicológicos implicados en la prevención de la victimización sexual y la elaboración de la App para la prevención de la violencia sexual, se proponen 6 publicaciones en inglés y una en español incluyendo en la propuesta los temas a abordar en cada artículo y especificando títulos orientativos. Se propone la participación en 5 congresos internacionales y 2 nacionales, así como 6 reuniones virtuales con colegas de la network que se plantea crear para construir redes de cooperación con investigadores de otros países. Impacto social También se propone para la diseminación de los resultados una seria de herramientas para la comunicación de los resultados y de la app a través de podcasts y reuniones virtuales con investigadores internacionales. También se plantea la potencialidad de transferencia con el desarrollo de un producto de interés comercial tanto a nivel nacional como internacional. En concreto se plantea la oportunidad de crear una spin-off para promover mejorar y aplicaciones de REVISE y la utilidad vinculada a la realidad virtual utilizada en esta app. Evaluando la eficacia de la aplicación REVISE, propuesta en esta investigación, se pretende aumentar las herramientas basadas en evidencia disponibles para una variedad de aplicaciones, como campañas educativas, psicoterapia especializada o programas de rehabilitación de agresores sexuales. La perspectiva de género está adecuadamente incorporada. En esta propuesta se tienen en cuenta los roles y estereotipos de género para construir un programa de RV que contribuya a contribuir masculinidades mas equitativas que permitirán que los hombres asuman su responsabilidad en la prevención de la violencia sexual y por cuestión de género promoviendo la empatía al permitir a través de RV que los hombres experimenten la victimización sexual desde la perspectiva de las mujeres.

4. Adecuación del presupuesto solicitado

En la propuesta se solicita un presupuesto total de costos directos de 106.850,00 ?, de los que la partida de mayor cuantía es la destinada a los gastos de personal de 66.890,00 ? destinado a la contratación de un informático para desarrollar la app y de un/a licenciado/a en psicología para llevar a cabo las entrevistas cualitativas. Para viajes y dietas se incluye un total de 12.260,00 ?, para otros gastos 26.700,00

? gastos de acogida de investigadores para generar la network y 20.000? que se destinarían para gastos derivados de las publicaciones en abierto, auditorias y gratificación a participantes. Para adquisición de inventariable 1.000,00 ?. El equipo está integrado con un numeroso numero de personal tanto en el equipo de investigación como de trabajo, incluido un ingeniero experto en RV, siendo la mayoría psicólogos/as que podrían hacerse cargo de las labores de evaluación por lo que no está suficientemente justificado los dos contratos propuestos. En relación a los gastos previstos de viajes y acogida de investigadores no son gastos que comprometen el desarrollo de la investigación propuesta, excepto el gasto de gratificación a participantes.

PARTE 2

OTROS ASPECTOS A CONSIDERAR

A) Capacidad Formativa

Se solicita la inclusión de un contrato predoctoral y se explicita un programa de actividades para el doctorando que se solicita incorporar al equipo, que se inicia con sus incorporación al programa en Salud y Psicologia de la Open Univesity of Catalonia. Se especifica la formación en metodología cuantitativa y cualitativa así como en el manejo de la RV. Asistencia a conferencia y encuentros científicos, elaboración de artículos también se tiene previsto que el/la candidata participe en estancias internacionales. No han dirigido ninguna tesis relacionada con el tema del proyecto y el equipo es un grupo de investigadores que no habían trabajado previamente en proyectos comunes, ni tienen publicaciones compartidas. Tienen solo una tesis defendida y esperan defender 4 en 2023, y enumeran una larga lista de doctorandos.

B) Aspectos relacionados con zonas polares o campañas oceanográficas

NO PROCEDE

C) Condiciones específicas para la ejecución de determinados proyectos

El estudio se registrará en el sitio web del Centro de Ciencia Abierta de la Open Science Foundation (http://osf.io/). El Centre for Open Science es una organización tecnológica sin ánimo de lucro con la misión de "aumentar la apertura, integridad y reproducibilidad de la investigación científica¿

INFORME-EXPERTO 2

PARTE 1

CRITERIOS DE EVALUACIÓN

1.Calidad y viabilidad de la propuesta

1.1 Calidad de la propuesta

El fin de esta propuesta se alinea con los proyectos orientados a la promoción de la Salud, en concreto aborda la prevención masculina de la victimización sexual (acoso) de las mujeres para impulsar la igualdad de género, reducir la desigualdad y potenciar la paz y justicia. Es innegable que el acoso sexual es una de las modalidades de violencia de género en el espacio público más normalizada que es vivida por muchas mujeres con resignación. Estas situaciones intimidatorias, hostiles y humillantes para las víctimas provocan miedo e inseguridad y afectan directamente a los derechos de las mujeres, limitando su movilidad, y libertad. El acoso sexual tiene consecuencias negativas de tipo cognitivo, emocional y conductual que pueden perdurar en el tiempo. La propuesta que trata de aplicar la realidad virtual a la prevención de los comportamientos sexuales agresivos hacia las mujeres es relevante ya que la mayoría de los programas actuales consiguen cambios en conocimientos y actitudes, pero su impacto sobre las conductas es muy limitado. Se trata de aplicar la perspectiva inmersiva en primera persona, de forma que parejas heterosexuales intercambiarán su identidad entre sí en un escenario de realidad virtual, a través de avatares con apariencias físicas similares a sus parejas, para mejorar la empatía y la perspectiva de las víctimas y aprender modelos de masculinidades sensibles al género. La hipótesis de partida no sólo es válida, sino que, además de ser original, está muy elaborada. Tiene en cuenta el bagaje científico en este campo de estudio para proponer nuevas perspectivas y consideraciones que permitan un avance en este ámbito del conocimiento. A tenor de lo indicado, el objetivo general es relevante ya que si los resultados obtenidos son favorables se podrá disponer de una herramienta actual que utiliza el potencial de la realidad virtual inmersiva de intercambio de identidades entre las parejas para promover cambios que ayuden a la prevención de la violencia sexual hacia las mujeres. Este objetivo se presenta de una manera realista, pero también es ambicioso al querer mostrar el potencial de una herramienta, que se encuentra en una fase incipiente, en un área de interés para la psicología. Aunque el proyecto no se presenta con carácter interdisciplinar ni multidisciplinar, en su desarrollo confluyen conocimientos derivados de la psicología e informática. De hecho, dentro del equipo de investigación, una de las personas es ingeniero informático.

1.2 Viabilidad de la propuesta

Los IPs cuentan con una media razonable de artículos publicados cada año en revistas especializadas (entre 3-5). Han participado en diversos proyectos de investigación y en algunos como IPs. Uno de los IPs ha creado y dirige el primer laboratorio de sexualidad humana en Latinoamérica, siendo uno de los resultados la adaptación de escalas sobre sexualidad. Parte de los esfuerzos de ambos IPs se han dirigido a la difusión y transferencia de sus resultados, bien publicando en revistas dirigidas a profesionales, interviniendo en medios de comunicación masivos o mejorando los recursos existentes en sexualidad. La producción científica del equipo es discreta, pero publican en revistas de impacto especializadas en su campo de estudio, menos algunos que hacen uso de revistas no especializadas de difusión masiva. Todos han participado en proyectos de investigación; una de las investigadoras cuenta con varias aplicaciones comerciales. Por tanto, las contribuciones del equipo garantizan en gran medida la viabilidad del proyecto. Los objetivos específicos son definidos de forma clara y precisa, su organización temporal es adecuada y van acorde con la duración del proyecto. La mayoría del equipo cuenta con experiencia en eHealth, si bien la trayectoria de algunos está más relacionada con los intereses del proyecto, en el caso de otros se aleja de estos intereses. La distribución de las tareas, así como la especialización y conocimientos de los miembros del equipo vinculados con la propuesta son un aval para la viabilidad del proyecto. Las actividades son descritas con rigor y precisión, a pesar de que el procedimiento es complejo. La metodología basada en un ensayo clínico aleatorizado es el más pertinente para el logro del objetivo general del proyecto. Algunas consideraciones son: 1) se desconoce dónde se va a reclutar a la muestra; 2) independientemente de la potencia estadística, 30 participantes por condición supone apostar por el número mínimo necesario; 3) no se justifica por qué el rango de edad va hasta los 39 años o cómo se va obtener información sobre la historia de acoso sexual o de violencia de género; 4) la medida conductual se basa solo en la medición eye tracking. La identificación de las cuestiones críticas y el plan de contingencia diseñado son correctos. Sin embargo, algunas de las propuestas del plan de contingencia no se concretizan y parecen difíciles de ejecutar.

2. Calidad y trayectoria de los componentes del proyecto

Ambos IPs tienen experiencia en eHEalth y en la aplicación de la Realidad Virtual en la promoción de la salud y la calidad de vida, específicamente uno de ellos en la temática del proyecto, la violencia sexual y la efectividad de protocolos de intervención psicológica. En el CV de ambos IPs consta la participación en un número importante de proyectos de investigación competitivos, de los cuales han sido IPs en varios de ellos. Por otra parte, en el registro del Ministerio de Ciencia e Innovación queda reflejado que ambos IPs han participado en 2 proyectos cada uno; uno de los IPs figura en un proyecto como coinvestigador principal. Uno de los proyectos de investigación versa sobre la victimización sexual femenina y algunos de ellos se basan en la aplicación de la realidad virtual. Un IP dirige un laboratorio sobre sexualidad humana que lo creó y el otro es coordinador de la International Society for Psychotherapy Research. A pesar de que las colaboraciones previas con las personas que forman el equipo son muy puntuales, los IPs presentan cierta capacidad de liderazgo. Si bien los miembros del equipo cuentan con un número de publicaciones discreto y han participado en algunos proyectos de investigación, la mayoría tienen experiencia y conocimientos en el uso de la realidad virtual y otras tecnologías en el campo de la salud principalmente. Algunas aportaciones importantes para la propuesta son las que han utilizado la realidad virtual en el estudio de la violencia de género en la pareja. La contribución de uno de los miembros del equipo es crucial ya que es el único ingeniero informático especializado en realidad aumentada, virtual y extendida, habiendo publicado un artículo que trata sobre la aplicación de la realidad virtual a las víctimas de acoso sexual. Por tanto, la experiencia y trayectoria previa de algunos de los miembros del equipo es coherente con los intereses del proyecto de investigación. Ambos IPs cuentan con estancias de investigación, aunque entre sus méritos más relevantes se refleja solo de forma parcial. Algunos miembros han realizado estancias internacionales y cuentan con algunas publicaciones donde hay coautores de universidades y centros extranjeros. Es de destacar que en el grupo de trabajo han incluido a tres investigadores internacionales de Ecuador, Portugal y

Suiza a los que se les ha asignado dentro del proyecto distintas tareas a desarrollar con la intención de poder adaptar el recurso por lo menos a alguno de estos países.

3. Impacto científico, económico y social esperado de los resultados

Si los resultados son esperanzadores, esta propuesta se traducirá en la elaboración de un recurso innovador basado en la aplicación de la realidad virtual para fomentar la implicación masculina en la prevención de los actos sexuales agresivos hacia las mujeres. Esta herramienta terapéutica y educativa junto con una guía de orientación podrá estar disponible para todos los profesionales que tratan de modificar los esquemas de género tradicionales y promover la igualdad de género. Esta aportación es relevante porque los recursos que existen para reducir los comportamientos sexistas y promover el desarrollo de nuevas masculinidades son escasos, y especialmente aquellos que utilizan este tipo de tecnología. El impacto social, que está bien definido en la memoria, se alinea con algunos de los objetivos del desarrollo sostenible. Sus aportaciones no sólo se ciñen a los actos agresivos cometidos en el ámbito público, sino que se pueden extender al ámbito laboral o al de las relaciones interpersonales. En definitiva, favorecerá el desarrollo de una sociedad más igualitaria, segura y respetuosa. El recurso derivado de este estudio podría tener un impacto económico importante, al reducir los costes derivados del tratamiento y de la incapacidad productiva de las víctimas de agresiones sexuales. Sin embargo, en el proyecto no se ha hecho ningún tipo de estimación basada en estudios económicos sobre los costes que suponen la victimización sexual de las mujeres. Se espera que este recurso se pueda transferir a la sociedad a través de la red profesional con la que cuenta el equipo como asociaciones en el campo de la psicoterapia, así como de agentes sociales y políticos incluidos en los departamentos del Ayuntamiento de Barcelona. El plan de producción científica consta de: realización de una tesis doctoral; publicación de 7 artículos científicos; 7 presentaciones a congresos; creación de una red de investigadores internacionales de las áreas de salud, humanidades e ingeniería; la aplicación y adaptación del recurso en países como Colombia y Ecuador. En el plan de divulgación se incluye la realización de una página web, podcasts, 3 talleres en centros de servicios sociales, educativos y organismos del tercer sector y reuniones virtuales. El plan de manejo de datos es completo; se podrá acceder a los datos en un repositorio fijo doi (Osf). La perspectiva de género está presente en todo el proyecto ya que es consustancial con el objetivo general de la propuesta.

4. Adecuación del presupuesto solicitado

El presupuesto previsto para la ejecución del proyecto es bastante elevado (106.850 ?). Todas las partidas están debidamente justificadas. Las partidas más cuantiosas son las relacionadas con los costes del personal: persona con licenciatura en psicología para la realización de las entrevistas en profundidad por parte de una persona ciega a la condición experimental y de un ingeniero superior para programar aplicaciones, mantenimiento de equipos y ajustes técnicos durante la fase piloto y la recogida de datos. Aunque se ha solicitado la inclusión en el programa de ayudas para contratos predoctorales para la formación de un doctor, estas tareas no podrían ser realizadas por esta persona.

PARTE 2

OTROS ASPECTOS A CONSIDERAR

A) Capacidad Formativa

La persona contratada se matriculará en el programa de doctorado en Psicología y Salud de la Universitat Oberta de Catalunya. Se desconoce si existe algún tipo de formación obligatoria para los estudiantes. Se plantea que colaborará en el proyecto para formarle en análisis cuantitativos y cualitativos, así como en la implementación avanzada de realidad virtual. Algunas de las tareas previstas son: participación en elaboración de artículos, asistencia a congresos, actividades de divulgación, webinars y talleres formativos. También están previstas dos estancias: una nacional en la Universidad de Granada que tiene un Labsex productivo o una estancia en la Universidad de Barcelona que investiga sobre temas de salud con realidad virtual; otra internacional que se realizará en Portugal con Pedro Nobre o Joana Carvalho (miembro del equipo de trabajo) que tienen experiencia en la investigación de la violencia sexual. No se concreta el tiempo de las estancias. El número de tesis defendidas bajo la dirección de los miembros del equipo no se indica, siendo muy escaso, lo que se atribuye a ser investigadores jóvenes, aunque la mayoría rondan los 40 años. Sin embargo, los dos IPs y los miembros del equipo tienen en curso 17 tesis que se defenderán entre el 2023 y 2028. Algunas de ellas guardan relación indirecta con el proyecto por la utilización de la realidad virtual o por abordar la salud sexual y el sexismo. Solo aportan información sobre el desarrollo científico y/o profesional de una doctora egresada que está contratada en la Universidad Internacional de la Rioja, cuenta con 6 artículos publicados y está realizando una estancia de 6 meses en USA. El contexto científico-tecnológico de la institución es prometedor. La Universitat Oberta de Catalunya cuenta con el Hub Interdisciplinar de Investigación e Innovación que se ha inaugurado recientemente (28/10/2022). Este nuevo hub reúne personal investigador y de apoyo, y ocho laboratorios; uno es el XR-Lab (laboratorio de tecnologías inmersivas) que contiene la principal infraestructura necesaria para el desarrollo de la propuesta. A pesar de que, en estos momentos, el equipo no cuenta con un grupo de doctores que hayan sido previamente formados, ya que prácticamente todos están realizando actualmente la tesis doctoral, y las colaboraciones previas entre ellos son escasas, tienen potencial para que la persona contratada reciba una formación adecuada y actualizada en análisis estadísticos y uso de nuevas tecnologías.

B) Aspectos relacionados con zonas polares o campañas oceanográficas

No aplica

C) Condiciones específicas para la ejecución de determinados proyectos

El procedimiento que van a llevar cabo en el estudio relacionado con estas condiciones específicas se explica de forma muy detallada. Se indica que el protocolo cumple con los criterios y estándares internacionales en ética que regula la investigación en Psicología. Además, se solicitará la aprobación del comité de ética de la Universitat Oberta de Catalunya. Se informará a lo participantes sobre todas las implicaciones de su participación en el proyecto, especialmente de las derivadas del uso de la realidad virtual, así como sobre sus derechos. La participación en el estudio requiere que los participantes firmen el consentimiento informado. Al final del estudio, los participantes serán informados sobre el propósito del experimento y monitoreado por cualquier efecto secundario a través de una entrevista semiestructurada realizada por un psicólogo. Asimismo, se enviará un correo electrónico de seguimiento después de dos semanas al participante con el fin de comprobar si han experimentado algún efecto secundario de haber estado involucrado en el escenario de realidad virtual. En una de las condiciones experimentales, los investigadores se asegurarán de que la escena de la realidad virtual no fomente actitudes sexistas inactivas o implícitas. Los participantes se les asignará de forma aleatoria a las condiciones y recibirán todos la misma retribución económica.

INFORME-EXPERTO 3

PARTE 1

CRITERIOS DE EVALUACIÓN

1.Calidad y viabilidad de la propuesta

1.1 Calidad de la propuesta

La propuesta es interesante y original, se adecúa perfectamente a las características y finalidad de la convocatoria, así como a la modalidad indicada y a la prioridad temática seleccionada. Es una propuesta relevante, de una temática de elevado interés (y necesidad) científica y social. Está perfectamente justificada en la memoria del proyecto, lo que supone un punto fuerte de la propuesta. La hipótesis de partida es válida y original, justificada e innovadora, a través del uso de la realidad virtual. Además, creo que la propuesta está muy bien redactada en la propuesta. Los objetivos están claramente definidos, son ambiciosos y relevantes, además de realistas, porque se pueden conseguir. En general, la propuesta es original y necesaria, lo que se deja ver perfectamente en la justificación.

1.2 Viabilidad de la propuesta

Como se ha manifestado en el apartado anterior, la propuesta es viable; es original, ambiciosa y, sobre todo, está muy bien justificada, tanto la necesidad de intervenir en el problema que se trata, como el procedimiento a seguir para hacerlo. Los resultados y contribuciones previos del equipo participante avalan también la viabilidad de la propuesta. Es un equipo amplio, como se comentara en el apartado siguiente, con una formación académica e investigadora complementaria. Volviendo al proyecto, los objetivos, general (ambicioso) y específicos se definen con claridad y precisión y facilitan el seguimiento de la memoria (y facilitarían valorar el grado de cumplimiento del proyecto, en caso de que se concediera). Son acordes con al duración del proyecto y con la composición del grupo, y siguen un orden lógico. La metodología y el procedimiento diseñados para cumplir esos objetivos son lógicos y adecuados. Se destacan las etapas críticas y, sobre todo, se presenta un plan de contingencia detallado en caso de que surgiera algún problema en la implementación de la propuesta. Este es un punto fuerte del proyecto, está muy cuidado.

2. Calidad y trayectoria de los componentes del proyecto

La propuesta cuenta con dos investigadores principales, con distinta formación y especialización investigadora, con currículums que se complementan, en el sentido de que uno de ellos es experto en intervención psicológica y el otro en el estudio de la sexualidad humana. Ambos parecen contar con la experiencia suficiente como para liderar una propuesta de estas características. El resto de miembros del equipo de investigación, que es amplio, permite aportar experiencia investigadora que complementa a los investigadores principales. Además de la intervención psicológica y de la sexualidad/victimización sexual, la otra pata fundamental de la propuesta es el uso de realidad virtual. Eso lo aportan otros miembros del equipo, con lo que sus aportaciones serán fundamentales (y, por tanto, la composición del equipo es adecuada).

3. Impacto científico, económico y social esperado de los resultados

La prevención de la victimización sexual es un tema transversal, que debería implicarnos e incluirnos a todo. Tiene, por tanto, un elevado componente social que hay que tener en cuenta y destacar. Si se hace ciencia para mejorar determinados problemas y, sobre todo, mejorar el bienestar y la calidad de vida de las personas, un proyecto de este tipo posee un impacto social relevante. En la propuesta se describe de forma adecuada este impacto, además de los resultados científico-técnicos que se desean obtener. Siete artículos (seis + uno, según explican los autores) es un plan ambicioso pero más que factible. Además, son resultados de investigación que incluyen a los propios participantes y que, durante la aplicación de la propuesta, permite mejorar ese bienestar de los participantes. Hay un plan adecuado de difusión/divulgación de los resultados, tanto a la comunidad científica como a la sociedad en general. Y también existe un adecuado plan de gestión de los datos de la investigación. La propuesta contempla, como elemento central, la dimensión de género. Creo que dado el tema de la propuesta, no merece la pena ni incidir en este aspecto, pues queda incorporado desde el origen. No obstante, el enfoque de género está descrito y justificado en la propuesta.

4. Adecuación del presupuesto solicitado

El presupuesto es adecuado para la implementación de la propuesta, aunque puede considerarse un tanto excesivo, sobre todo en algunas partidas (20.700 euros para "otros gastos", 12.260 euros para asistencia a congresos, casi 70 mil euros en personal,...). Creo sinceramente que se podría llevar a cabo con la mitad del presupuesto solicitado, ya que el gasto en logística (realidad virtual, participantes) se podría cumplir. Es el único apartado que deja algunas dudas.

PARTE 2

OTROS ASPECTOS A CONSIDERAR

A) Capacidad Formativa

Se solicita la inclusión del proyecto en el programa de formación, que queda descrito de forma adecuada dentro de la propuesta solicitada. El equipo de investigación parece poseer la capacidad formativa suficiente para ello.

B) Aspectos relacionados con zonas polares o campañas oceanográficas

No aplica.

C) Condiciones específicas para la ejecución de determinados proyectos

No aplica.

INFORME-EXPERTO 4

PARTE 1

CRITERIOS DE EVALUACIÓN

1.Calidad y viabilidad de la propuesta

1.1 Calidad de la propuesta

El proyecto se adecua a las características y finalidad de la convocatoria, con una excelente propuesta de generación de conocimiento en un ámbito de investigación relevante socialmente. Así mismo, el proyecto contempla la posibilidad de contar con la participación de grupos de investigación innovadores capaces de generar sinergias entre diferentes enfoques científico-técnicos, y promover avances en el conocimiento científico para incidir en el ODS 3, ODS5, ODS10 y ODS16. Los objetivos del proyecto son acordes al área temática seleccionada, salud. La hipótesis general del proyecto es innovadora y ambiciosa, y permitiría un gran avance en el conocimiento sobre procesos interpersonales necesarios para la intervención en situación de violencia contra las mujeres, en este caso, situaciones de acoso, pero sin duda extrapolables a otras situaciones similares y de gran relevancia social. EL proyecto trata de probar la hipótesis de que una novedosa intervención inmersiva de RV, REVISE, tendrá un impacto significativo en el comportamiento de los hombres y en su comprensión crítica de la violencia sexual y de género. Concretamente esperan que la perspectiva encarnada como víctima de acoso sexual mejorará la empatía de los participantes y reducirá las actitudes sexistas. Los objetivos están claramente definidos, así como el procedimiento y metodología para su consecución, todos ellos definidos de manera concreta y ajustados a los tiempos de ejecución del proyecto (4 años) indicando en cada momento las personas responsables de su desarrollo y ejecución. Pese a que no indicar un enfoque interdisciplinar, a lo largo de los objetivos e hipótesis, si se indica la idoneidad de cada uno de los investigadores en las diferentes tareas de investigación, así como en las potenciales actividades de difusión y transferencia de resultados. Forman un equipo con el conocimiento y experiencia necesaria para desarrollar el plan propuesto.

1.2 Viabilidad de la propuesta

La evidencia empírica que aporta en el documento subraya la importancia de la perspectiva inmersiva en primera persona para aumentar el poder transformador de la experiencia de RV. Un miembro del equipo de investigación (Seinfeld et al., 2018), demostró su eficacia en la modificación de procesos socioperceptivos como el reconocimiento de emociones, que se cree que subyacen a los comportamientos agresivos. Así mismo la utilidad de la RV en el estudio de comportamientos violentos ha sido demostrados en estudios previos. Los objetivos específicos están descritos de manera concreta y ajustados al tiempo de ejecución del proyecto, así como vinculados a personas concretas responsables de los mismos, acorde a la formación de los miembros del equipo. Proponen alcanzar 7 objetivos específicos, contemplando la participación, no solo de los participantes objeto del estudio (parejas de jóvenes heterosexuales), sino la colaboración de mujeres víctimas de acoso sexual y profesionales que asisten a agresores masculinos (objetivo 2), así como a centros específicos orientados a la comunidad. Describen el proceso de selección de la muestra de acuerdo al diseño, así como una excelente descripción de los instrumentos, diseño y procedimiento. Incluye un exhaustivo detalle del análisis de los datos y tratamiento de los mismos de acuerdo con los estándares científicos. Se describe el procedimiento de tratamiento de datos, así como el protocolo del comité de ética solicitado para la propuesta de investigación. La característica diferencial que hace novedosa e innovadora la propuesta es el enfoque de métodos mixtos midiendo el impacto de la intervención con RV en 3 niveles: a) cuestionarios autoinformados para observar los cambios en variables clave relacionadas con las habilidades de empatía, los esquemas de roles de género y las características de las relaciones. b) medidas fisiológicas- para medir la activación emocional y el seguimiento ocular para examinar los cambios en los comportamientos de objetivación sexual antes y después de la exposición al acoso sexual mediante RV; y c) entrevistas cualitativas sobre el impacto de la intervención en la comprensión de los roles de género, y su relación con el acoso sexual y la violencia sexual. Considero muy positivo el hecho de incorporar a la pareja para dar su feedback cuando la pareja experimenta el acoso en primera persona. Es una excelente manera de activar la empatía en los hombres acerca de las situaciones de discriminación, acoso y violencia que sufren las mujeres en su dia a dia por el hecho de ser mujeres. Cuenta además con un equipo multidisciplinar sólido y con experiencia previa en el tema de estudio, e incorpora planes de acción alternativos en caso de verse afectados por algún tipo de improviso que pudiera poner en riesgo la viabilidad de la propuesta. Plan de emergencia incluido de manera que se pueda garantizar la viabilidad del proyecto en todo momento.

2. Calidad y trayectoria de los componentes del proyecto

Los IPs del proyecto, tienen experiencia previa en este tipo de investigación y ambos son miembros de un grupo de investigación emergente (SGR2021). Sus áreas de especialización se combinan en la concepción de esta propuesta de proyecto. Los IP tienen amplia experiencia en la realización de ensayos clínicos en entornos de salud mental e intervenciones psicológicas, así como en liderar proyectos de investigación, algunos de ellos sobre RV. Sus trabajos previos han demostrado cómo la personalización de la RV para explorar las percepciones de uno mismo y de los demás puede fomentar el compromiso en las intervenciones psicológicas con jóvenes. También han publicado varios artículos sobre terapia de pareja y familiar, en los que concibe y se pone a prueba intervenciones para mejorar las relaciones. El segundo IP además tiene formación específica en el estudio de la conducta sexual. Así mismo el resto del equipo, tiene experiencia en el estudio de tecnologías inmersivas y trabajos previos relacionados con la temática que se aborda en este proyecto, con numerosos artículos publicados, participación en proyectos de investigación, nacionales e internacionales, así como varias tesis dirigidas relacionadas con el tema que garantizan la viabilidad de la propuesta. En cuanto a la internalización, se prevé la elaboración de al menos 6 artículos de investigación que enviara a revistas internacionales indexadas: BMJ Open, Family Process, Scientific Reports, entre otras. Han previsto a redes de cooperación con investigadores e instituciones colombianas y ecuatorianas que puedan gestionar recursos de sus respectivos Ministerios de Ciencia y/o Embajadas de la Unión Europea para implementar el proyecto.

3. Impacto científico, económico y social esperado de los resultados

La propuesta tiene un alto interés científico, que radica en aportar pruebas para una aplicación de RV inmersiva, REVISE, como herramienta transformadora del compromiso masculino en la prevención de la violencia sexual. Una propuesta con posibilidad de transferencia ya que REVISE podrá ser de interés no solo en el ámbito académico, sino también en organizaciones científicas-asociaciones profesionales y organismos especializados en la promoción de la igualdad de género. Proponen la creación de una red de investigadores de las áreas de la salud, las humanidades y la ingeniería, para tecnológicas aplicadas a la promoción de la salud sexual. En cuanto al impacto social, el desarrollo de la propuesta se enmarca en la agenda global propuesta en los Objetivos de Desarrollo Sostenible, ODS 3 y ODS10 fundamentalmente. Incluyen un excelente plan de ejecución del proyecto contando con el apoyo de asociaciones profesionales del ámbito de la psicoterapia. Cuentan con el apoyo de agentes sociales y políticos, incluidos los departamentos del Ayuntamiento de Barcelona: concejalía de Feminismos y LGTBI, concejalía de Juventud, y la red de PuntInfoJove, y el apoyo de colectivos relevantes de atención a víctimas de agresiones sexuales. Uso de página web y podcats para mostrar las actividades del proyecto y su desarrollo, 3 talleres de difusión de conocimientos (dos en centros educativos y de servicios sociales españoles, y otra jornada con organizaciones del tercer sector y responsables políticos) y la participación en conferencias internacionales y nacionales relacionados con el proyecto. La perspectiva de género está en el eje central de la propuesta de investigación y presente en todas las etapas del ciclo de investigación, desde el problema a tratar (violencia contra las mujeres) hasta la difusión y el desarrollo tecnológico derivado de los resultados. Pretenden el desarrollar y validar el uso de tecnología de RV que permita el intercambio de géneros para que los hombres experimenten la victimización sexual desde la perspectiva de una mujer, y favorecer así la construcción de masculinidades más equitativas desde el punto de vista del género, lo que daría lugar a una mayor empatía y comprensión de la violencia sexual y de género por parte de los hombres. Los resultados de la propuesta pueden materializarse en intervención de carácter educativo, preventivo y terapéutico, promoviendo un mayor compromiso de los hombres en la prevención de la victimización sexual.

4. Adecuación del presupuesto solicitado

Presentan un presupuesto detallado y ajustado a los objetivos desarrollados en el proyecto. Indican de manera pormenorizada las cuantías en cada partida de manera desglosada. Una parte importante del presupuesto está destinado a la creación de la aplicación REVISE y todas las funciones que necesitan para su optimo funcionamiento y desarrollo del proyecto. Unido a esto, la necesidad de contratación de personal especializado en su uso (un ingeniero informático y un programador). Para llevar a cabo los objetivos 5 y 6 (entrevistas cualitativas) estiman 400 horas de asistentes de investigación psicólogos externos (coste estimado: 8.093 euros) cuya tarea consistirá en realizar y grabar entrevistas en profundidad con las 100 parejas (200 personas) que participan en el estudio. Prevén compensación económica para los participantes y reducir de esta manera la falta de datos en las evaluaciones de seguimiento. Incluyen coste de publicación, auditoría y actividades de difusión y creación de redes.

PARTE 2

OTROS ASPECTOS A CONSIDERAR

A) Capacidad Formativa

El equipo compuesto, en su mayoría, por investigadores con experiencia en la dirección de tesis doctorales, dirección de proyectos de investigación tanto nacionales como internacionales, así como estancias de investigación internacional. El personal en formación estará inscrito en el programa de doctorado en Salud y Psicología, y participará en la implementación del proyecto. Para ello contará con la formación en análisis de datos cuantitativos y cualitativos, así como en la implementación avanzada de la RV. Se espera que participe en la publicación de artículos, asistencia a conferencias para presentar los resultados derivados del proyecto y participar en actividades de divulgación para un público general y especializado. Prevén una estancia en la Universidad nacional así como estancias internacionales en Portugal (Dr Pedro Nobre o Joana Carvalho) con investigadores con experiencia en investigación sobre violencia sexual. El personal en formación también asistirá a diferentes webinars y talleres formativos y en sus últimos años de formación contribuirá a estos seminarios web. Aunque su trayectoria en común es corta, se trata de un equipo de jóvenes investigadores, con amplia experiencia en la formación doctoral. Siete de los miembros del equipo de investigación tienen experiencia en dirección de tesis, una de ellas defendida en 2023, relacionada con el proyecto, y prevista la defensa de cuatro nuevas tesis (una de ellas ya depositada). Tienen 6 tesis en curso directamente relacionadas con el proyecto. Una egresada defendió su doctorado en 2020 bajo la dirección de Alejandro Guillén y actualmente es CD en la Universidad Internacional de La Rioja (UNIR). Cuenta con 6 artículos en revistas indexadas en el SJR con un índice h de 3, y 29 citas. Actualmente está realizando unas prácticas en Estados Unidos. Por su parte, la Universitat Oberta de Catalunya cuenta con un recién inaugurado centro Interdisciplinar de Investigación e Innovación que reúne personal investigador y de apoyo, y ocho laboratorios, uno de los cuales es el XR-Lab (laboratorio de tecnologías inmersivas). El XR-Lab contiene la principal infraestructura técnica necesaria para el desarrollo de la propuesta. El centro ofrece apoyo y asesoramiento técnico para experimentos al personal investigador, alojamiento de servidores para grupos de investigación y proyectos de experimentación, provisión de servidores en la nube para grupos de investigación

B) Aspectos relacionados con zonas polares o campañas oceanográficas

No procede

C) Condiciones específicas para la ejecución de determinados proyectos

No procede
